# House dust mites as potential carriers for IgE sensitization to bacterial antigens

**DOI:** 10.1111/all.13260

**Published:** 2017-09-07

**Authors:** S. Dzoro, I. Mittermann, Y. Resch‐Marat, S. Vrtala, M. Nehr, A. M. Hirschl, G. Wikberg, L. Lundeberg, C. Johansson, A. Scheynius, R. Valenta

**Affiliations:** ^1^ Department of Pathophysiology and Allergy Research Centre for Pathophysiology, Infectiology and Immunology Medical University of Vienna Vienna Austria; ^2^ Division of Clinical Microbiology Clinical Institute of Laboratory Medicine Medical University of Vienna Vienna Austria; ^3^ Dermatology and Venereology Unit Karolinska University Hospital Stockholm Sweden; ^4^ Department of Clinical Science and Education Karolinska Institutet Stockholm Sweden; ^5^ Sachs’ Children and Youth Hospital Södersjukhuset Stockholm Sweden

**Keywords:** allergens and epitopes, atopic dermatitis, basic mechanisms, house dust mites

## Abstract

**Background:**

IgE reactivity to antigens from Gram‐positive and Gram‐negative bacteria is common in patients suffering from respiratory and skin manifestations of allergy, but the routes and mechanisms of sensitization are not fully understood. The analysis of the genome, transcriptome and microbiome of house dust mites (HDM) has shown that *Staphylococcus aureus* (*S. aureus*) and *Escherichia coli* (*E. coli*) species are abundant bacteria within the HDM microbiome. Therefore, our aim was to investigate whether HDM are carriers of bacterial antigens leading to IgE sensitization in patients suffering from atopic dermatitis.

**Methods:**

Plasma samples from patients with AD (n = 179) were analysed for IgE reactivity to a comprehensive panel of microarrayed HDM allergen molecules and to *S. aureus* and *E. coli* by IgE immunoblotting. Antibodies specific for *S. aureus* and *E. coli* antigens were tested for reactivity to nitrocellulose‐blotted extract from purified HDM bodies, and the IgE‐reactive antigens were detected by IgE immunoblot inhibition experiments. IgE antibodies directed to bacterial antigens in HDM were quantified by IgE ImmunoCAP™ inhibition experiments.

**Results:**

IgE reactivity to bacterial antigens was significantly more frequent in patients with AD sensitized to HDM than in AD patients without HDM sensitization. *S. aureus* and *E. coli* antigens were detected in immune‐blotted HDM extract, and the presence of IgE‐reactive antigens in HDM was demonstrated by qualitative and quantitative IgE inhibition experiments.

**Conclusion:**

House dust mites (HDM) may serve as carriers of bacteria responsible for the induction of IgE sensitization to microbial antigens.

**Abbreviations**
AD: atopic dermatitis
Der p: *Dermatophagoides pteronyssinus*
HDM: house dust mites
IgE: immunoglobulin E
IgG: immunoglobulin G
ISAC: immuno solid‐phase allergen chip
ISU: immuno solid‐phase allergen chip standardized units
kU/L: kilo units per litre
kU_A_/L: kilo units antigen per litre
MeDALL: mechanisms for the development of allergies
OD: optical density
SCORAD: scoring atopic dermatitis

## 1 INTRODUCTION

Already in 1932, Cooke suggested that allergic sensitization to bacteria may play a role in asthma.^1^ Since then, IgE sensitization to bacterial antigens has been reported for different manifestations of allergy. Several studies have observed IgE sensitization to bacterial antigens in children suffering from asthma.^2^, ^3^, ^4^ In particular, IgE antibodies directed against enterotoxins from *Staphylococcus aureus* (*S. aureus*) were found to be associated with severe forms of asthma^5^, ^6^ and recently Staphylococcal serine protease‐like proteins were suggested as pacemakers of allergic airway reactions to *S. aureus*.^7^

The occurrence of IgE to bacterial antigens was also observed in atopic dermatitis (AD) patients as early as 1981.^8^, ^9^, ^10^, ^11^, ^12^ Anti‐Staphylococcal IgE has been found in atopic dermatitis patients with or without cutaneous Staphylococcal infection,^8^, ^9^, ^10^, ^11^, ^12^ and IgE reactivity against various antigens such as Staphylococcal exotoxins^13^ and *S. aureus* fibronectin‐binding protein^14^ has been reported. In addition to IgE reactivity against Staphylococcal antigens, IgE reactivity to *Escherichia coli* (*E. coli*) antigens and other bacteria of the gut has been detected in about one‐third of patients with AD.^15^ These findings are unexpected, considering that *S. aureus* typically induces Th1 or Th17 rather than Th2 immune responses, and nonpathogenic *E. coli* is associated with immune tolerance rather than with allergic sensitization.^16^, ^17^

*Staphylococcus aureus* (*S. aureus*) is present in the skin of patients with AD who frequently suffer from *S. aureus* superinfections, and it has therefore been suggested that sensitization occurs via the skin.^17^ By contrast, *E. coli* and other bacteria (eg, *Haemophilus influenza*) which have been reported as sources for IgE‐reactive antigens^3^, ^4^, ^15^ can be found mainly in the gut and in the respiratory tract, suggesting that other routes and mechanisms of allergic sensitization may be important. Recently, the genome, transcriptome and microbiome of house dust mites (HDM) one of the most frequent and potent allergen sources have been reported.^18^ More than 50% of atopic individuals have allergic sensitization to HDM, particularly in environments with favourable conditions for mite proliferation,^19^, ^20^, ^21^ and sensitization to HDM is common in patients suffering from respiratory and skin manifestations.^20^, ^22^, ^23^ Interestingly, the microbiome of *Dermatophagoides farinae* comprises a variety of Gram‐positive and Gram‐negative bacteria, among them *S. aureus*,* E. coli* and *Enterobacteria*.^18^ The description of the HDM microbiome has contributed to a growing body of evidence highlighting that HDM not only contain allergens but also other components such as bacterial adjuvant compounds which can activate the innate immune system.^24^, ^25^

Here, we hypothesized that HDM may have another hitherto unknown role in allergic sensitization. In fact, we investigated whether HDM are carriers of microbial antigens that could cause a Th2 type adaptive immune response to bacterial antigens. To study this hypothesis, several lines of investigations were pursued. First, we studied the occurrence of IgE sensitization to HDM and bacterial antigens from *S. aureus* and *E. coli*, in a group of patients with AD from Sweden where sensitization to HDM is expected to be less common, as a result of the cold climate.^26^ This allowed us to evaluate whether sensitization to bacterial antigens is more frequent among HDM‐sensitized patients. In addition, we investigated the presence of bacterial antigens in extracts prepared from purified HDM bodies. Finally, we were able to demonstrate that HDM contain IgE‐reactive bacterial antigens using sera from patients sensitized to *S. aureus* and *E. coli*.

## 2 METHODS

### 2.1 Allergen extracts from house dust mites, *S. aureus* and *E. coli*

An allergen extract from HDM was prepared using purified bodies of *Dermatophagoides pteronyssinus*, purchased from Allergon AB (Ängelholm, Sweden). Purified mite bodies were purchased to minimize the effect of faecal contamination in our analysis. Protein extracts were prepared by adding 5 mL of Laemmli sodium dodecyl sulphate (SDS) sample buffer (62.5 mmol/L Tris‐HCl pH 6.8; 2.5% SDS; 0.002% bromophenol blue; 0.7135 mol/L (5%) β‐mercaptoethanol; 10% glycerol)^27^ to 0.3 g of purified mites and sonicating using an Ultra Turrax (IKA Labortechnik, Staufen, Germany). The homogenate was then incubated at 95°C for 10 minutes followed by centrifugation at 14 000 × *g*, and supernatants were stored at −20°C until use.

Clinical isolates of *S. aureus* strain ATCC25923, and *E. coli* strain ATCC25922, were obtained as live cultures from the Leibniz Institute German collection of microorganisms and cell cultures (DSMZ‐Deutsche Sammlung von Mikroorganismen und Zellkulturen GmbH, Braunschweig, Germany). Isolates were grown overnight at 37°C in 500 ml Brain Heart Infusion Medium and then centrifuged at 3220 × *g* to harvest bacterial cells. Pellets were washed twice in phosphate‐buffered saline (PBS) and inactivated by 3 cycles (one minute each) of alternate heating (95°C) and freezing (liquid nitrogen). Inactivated bacterial cells were resuspended in 5 mL PBS and sonicated using an Ultra Turrax (IKA Labortechnik). Protein concentrations in the extracts were analysed by the Pierce™ BCA Protein Assay (Thermo Scientific, Rockford, IL, USA).

### 2.2 Patients’ sera and IgE serology

Plasma samples from a cohort of 319 Swedish adults aged between 18 and 65 years (median age 28 years)^28^ were analysed for total plasma IgE levels, IgE reactivity to HDM allergens and HDM extract and IgE reactivity to *S. aureus* extract and *E. coli* extract. The cohort consisted of 179 patients with moderate to severe AD (001AD‐179AD), 43 patients with seborrhoeic eczema (01S‐43S) and 97 nonatopic individuals without clinical symptoms of skin disease or history of allergy (01N‐97N). Participants were recruited at the Dermatology and Venereology Unit at Karolinska University Hospital, Stockholm, Sweden,^28^ where they were examined by a dermatologist. Where applicable, the severity of atopic skin disease was determined by the SCORAD and graded as moderate or severe, depending on the extent and intensity of the inflammatory lesions.^29^, ^30^ The Swedish AD patients were frequently sensitized to several environmental allergens such as allergens from cat (55%), grass (54%), birch (52%) and dog (37%) as determined using microarrayed allergens.^28^ Data on atopic conditions such as asthma and rhinitis were collected by anamnesis (medical history). The study was approved by the regional ethical review board in Stockholm, and written informed consent was obtained from all participants.

For IgE immunoblotting and IgE inhibition experiments requiring large volumes of serum, we included additional samples from well‐characterized allergic patients (A1‐A6) (Table S1) with known IgE reactivity to bacterial antigens.^15^ Serum samples were analysed in an anonymized manner with approval of the ethics committee of the Medical University of Vienna, Austria (EK1641/2014).

All work was conducted in compliance with the Declaration of Helsinki code of ethics for studies on human subjects.

### 2.3 Specific rabbit antibodies

Rabbit immunizations were conducted at the Charles River Laboratories in France, internationally accredited by the Association for Assessment and Accreditation of Laboratory Animal Care (AAALAC). Immunization procedures were carried out in compliance with the directive 2010/63/EU of the European Parliament and of the council of 22 September 2010, on the protection of animals used for scientific purposes. Antisera specific for *E. coli* or *S. aureus* were raised by subcutaneous immunization of rabbits with 3 injections, each containing 400 μg bacterial proteins (*S. aureus* or *E. coli*). Complete Freund's adjuvant was used in the first injections, followed by 2 subsequent booster injections in incomplete adjuvant after 4 weeks and after an additional 3 weeks, respectively.

In addition, a rabbit antiserum capable of detecting over 300 low to high molecular weight proteins from *E. coli* was obtained from Rockland Immunochemicals (Limerick, PA, USA). For purposes of this work, the anti‐*E. coli* antiserum raised from rabbit immunized with *E. coli* extract is referred to as anti‐*E. coli.1*, and the purchased anti‐*E. coli* antiserum is referred to as anti‐*E. coli.2*. Preimmune serum was not available for the (commercial) anti‐*E. coli*.*2*.

Rabbit antisera specific for nDer p 1, rDer p 2, rDer p 5, rDer p 7, rDer p 11, rDer p 14, rDer p 21, rDer p 23 or clone 16 were obtained by immunization with the purified allergens^31^, ^32^, ^33^, ^34^, ^35^, ^36^ (Valenta & Curin, unpublished). Preimmune serum was available for rabbits immunized with rDer p 2, rDer p 7, rDer p 10, rDer p 11, rDer p 14 and rDer p 23 but not for rabbits immunized against nDer p 1, rDer p 5, rDer p 21 or clone 16.

### 2.4 Detection and measurement of specific IgE and IgG antibodies

IgG and IgE antibodies specific for *S. aureus* and *E. coli* were detected by immunoblotting.^15^ Bacterial extracts were added to Laemmli SDS sample buffer,^27^ boiled for 5 minutes and separated by SDS‐PAGE on a 12.5% SDS polyacrylamide gel.^37^ As HDM extracts were already prepared in Laemmli SDS sample buffer, the HDM protein extracts were loaded directly on 12.5% SDS polyacrylamide gels for electrophoretic separation. Aliquots of 50 μg, of each of the extracts were loaded per centimetre of a preparative gel, and a prestained protein ladder (PageRuler™ Plus, Thermo Scientific) was used as a molecular weight marker. The separated proteins were transferred to a nitrocellulose membrane by electroblotting in transfer buffer (25 mmol/L tris base; 190 mmol/L glycine; 20% methanol).^38^ Membranes were stained with Ponceau S dye to visualize protein bands and allowed to air dry. Strips were cut from the membranes for immunoblotting.

For detection of rabbit IgG binding, strips were washed, blocked and incubated with rabbit preimmune or immune sera diluted 1/5000 in gold buffer. The anti‐*E. coli.2* antiserum was diluted 1/1000 in gold buffer. Bound rabbit IgG was detected with I^125^ donkey anti‐rabbit IgG antibodies (1/10 000) (Perkin Elmer, Waltham, MA, USA), and signals were visualized by autoradiography.^39^

For detection of human IgE binding, strips were washed and blocked in gold buffer (52.8 mmol/L Na_2_HPO_4_; 8 mmol/L NaH_2_PO_4_; 0.5% bovine serum albumin (BSA); 0.5% tween 20; 0.05% NaN_3_) and incubated overnight at 4°C, with plasma diluted 1/10. After the incubation with plasma, strips were washed, and bound IgE was detected with I^125^ labelled anti‐human IgE antibodies at a 1/10 dilution (Demeditec Diagnostics, Kiel, Germany). Signals were visualized by autoradiography. Positive IgE reactivities were defined by the presence of at least one clearly visible band on the autoradiograph, distinct from any background bands seen on the strip incubated with plasma/sera from nonallergic subjects and buffer alone (buffer control).

IgE reactivity to a comprehensive panel of purified Der p allergens (nDer p 1, rDer p 2, rDer p 4, rDer p 5, rDer p 7, rDer p 10, rDer p 11, rDer p 14, rDer p 15, rDer p 18, rDer p 21, rDer p 23, clone 16) was analysed with the customized allergen chip (Phadia Multiplexing, Thermo Fisher Scientific, Vienna, Austria) using ImmunoCAP ISAC (MeDALL) technology as previously described.^40^, ^41^, ^42^ The cut‐off to be considered positive for IgE sensitization to any of the individual Der p allergens was set at 0.1 ISU. In addition, IgE specific to *D. pteronyssinus* extract was quantified using the d1 ImmunoCAP™ (Thermo Fisher Scientific, Uppsala, Sweden) and the cut‐off for IgE sensitization was set at 0.1 kU_A_/L.

Total plasma IgE was quantified by the total IgE ImmunoCAP™ (range: 2‐5000 kU/L, Thermo Fisher Scientific). Samples with total IgE levels above 5000 kU/L were diluted in ImmunoCAP sample buffer to obtain the actual total IgE levels.

### 2.5 Immunoblot and ImmunoCAP IgE inhibition assays

In IgE immunoblot inhibition experiments, 1/10 diluted plasma/serum samples were preincubated overnight (4°C) with 200 μg of *E. coli* extract or for control purposes, with 0.5% bovine serum albumin and then tested for IgE reactivity to nitrocellulose‐blotted HDM or *E. coli* extract.^39^ The ImageJ software^43^ was used to analyse and quantify the intensity of bands observed on the autoradiographs. The reduction in density of bands from samples preincubated with bovine serum albumin compared to samples preincubated with *E. coli* extract was expressed as % inhibition of IgE binding.

Quantitative ImmunoCAP™ inhibitions were performed by preincubating 100 μL of plasma/sera with 100 μg of *E. coli* extract in 50 μL PBS or with 50 μL of PBS alone overnight at 4°C. Preadsorbed patient samples were then analysed for Der p‐specific IgE reactivity on the ImmunoCAP™ 250 auto analyser (Phadia, Portage, MI, USA). Resulting IgE levels were corrected for dilution (factor 1.5). The percentages of inhibition of IgE binding to the Der p ImmunoCAP™ were calculated by comparing results from samples preincubated with *E. coli* extract (inhibited) with results from samples preincubated with PBS only (uninhibited), using the formula: (uninhibited‐inhibited)/uninhibited × 100.

### 2.6 Statistical analysis

Statistical analysis was performed using GraphPad Prism version 6.07 for Windows, GraphPad Software, La Jolla, CA, USA. Contingency data were analysed by Fisher's exact test, and the Mann‐Whitney test was used for comparison of group medians. Odds ratio and risk ratio estimates, differences in proportions, differences in medians and the corresponding 95% confidence intervals were calculated. All calculated probability (*P*) values were two‐tailed, and *P* values <.05 were considered statistically significant. Venn diagrams were generated using the Biovenn Web application http://www.cmbi.ru.nl/cdd/biovenn/.^44^

## 3 RESULTS

### 3.1 Approximately one‐third of Swedish AD patients show IgE reactivity to bacterial antigens

**Figure 1:**
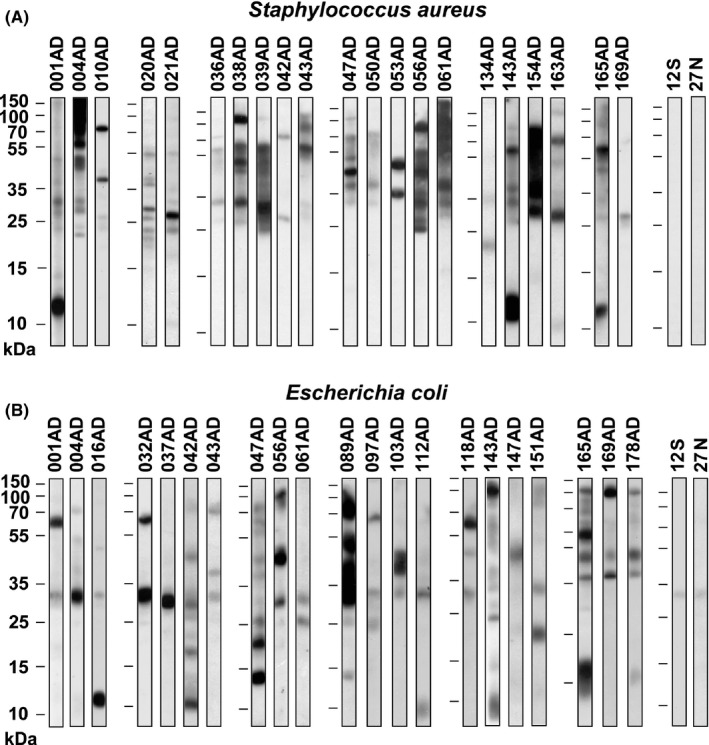
Representative IgE reactivity profiles to bacterial antigens in Swedish AD patients. IgE binding to nitrocellulose‐blotted (A) *Staphylococcus aureus* (*S. aureus*) extract or (B) *Escherichia coli* (*E. coli*) extract, observed in patients with AD but not in seborrhoeic eczema patients (S) or non‐atopic individuals (N) (negative controls). Molecular weights (kDa) are indicated on the left margins

Figure 1A,B shows representative IgE reactivities to nitrocellulose‐blotted *E. coli* and *S. aureus* extracts, respectively, which were observed exclusively in the 179 patients with AD but not in patients with seborrhoeic eczema (n = 43) or in nonatopic individuals (n = 97). Total plasma IgE levels ranged from <2 kU/L to 390 kU/L for the seborrhoeic eczema (SE) patients, up to 230 kU/L for the nonatopic controls and up to 15 100 kU/L among the patients with AD. Sixty‐one per cent of the patients with AD (n = 110) had IgE levels comparable to the control groups (ie, ≤350 kU/L).

Patients with AD showed IgE reactivity to several different bands in *S. aureus* and *E. coli* extracts as exemplified in Figure 1. However, no major IgE‐reactive antigens recognized by more than 50% of the patients could be identified when the complete immunoblot data were evaluated (data not shown). IgE reactivity to at least one band in *S. aureus* and *E. coli* was observed in 21% (n = 38) and 25% (n = 45) of the 179 patients with AD, respectively. Thirty‐three per cent of the patients with AD (n = 59) showed IgE reactivity to at least one of the bacterial extracts, and 13% (n = 24) had IgE to both bacteria. Sixty‐three per cent of the participants who had IgE to *S. aureus* also had IgE to *E. coli* and 53% of those who had IgE to *E. coli* also had IgE to *S. aureus*. We thus noted that the frequency of IgE sensitization to these bacterial antigens was lower in the Swedish AD patients (ie, 33%) as compared to that observed for Austrian and German AD patients earlier (ie, almost 40%).^15^

### 3.2 Low prevalence of IgE sensitization to HDM allergens in Swedish AD patients

The frequency of IgE sensitization to HDM allergens was 25% among the Swedish AD patients, when measured by the allergen chip (Table 1).^28^ Sensitization to HDM allergens was therefore considerably lower in the Swedish AD patients than in the Austrian and German AD patients (ie, more than 50%) who had been analysed for IgE reactivity to bacterial antigens earlier.^15^

**Table 1:** Frequencies of IgE reactivity to bacterial antigens in HDM‐sensitized and HDM‐nonsensitized Swedish AD patients

IgE specific to:	HDM allergens^a^		Odds ratio (95% CI)	*P*‐value
	Positive (n = 45) n (%)	Negative (n = 134) n (%)		
*S. aureus* b (n = 38)	18 (40)	20 (15)	3.8 (1.772‐8.148)	.0007
*E. coli* c (n = 45)	26 (58)	19 (14)	8.3 (3.853‐17.810)	<.0001
*E. coli* and/or *S. aureus* (n = 59)	30 (67)	29 (22)	7.2 (3.441‐15.240)	<.0001

CI, confidence interval.

a Der p allergens on the MeDALL chip (nDer p 1, rDer p 2, rDer p 4, rDer p 5, rDer p 7, rDer p 10, rDer p 11, rDer p 14, rDer p 15, rDer p 18, rDer p 21, rDer p 23, clone 16), positive cut‐off ≥0.1 ISU IgE to at least 1 of the 13 allergens.

b *Staphylococcus aureus* ATCC25923 extract.

c *Escherichia coli* ATCC25922 extract.

Age and gender distribution did not differ significantly between Swedish AD participants with HDM allergy and those without HDM allergy (Table S2A). However, HDM‐sensitized AD patients had significantly higher levels of total IgE than HDM‐nonsensitized AD patients (Figure S1, Table S2A).

### 3.3 The frequency of IgE sensitization to bacterial antigens is significantly higher in patients with AD sensitized to HDM

**Figure 2:**
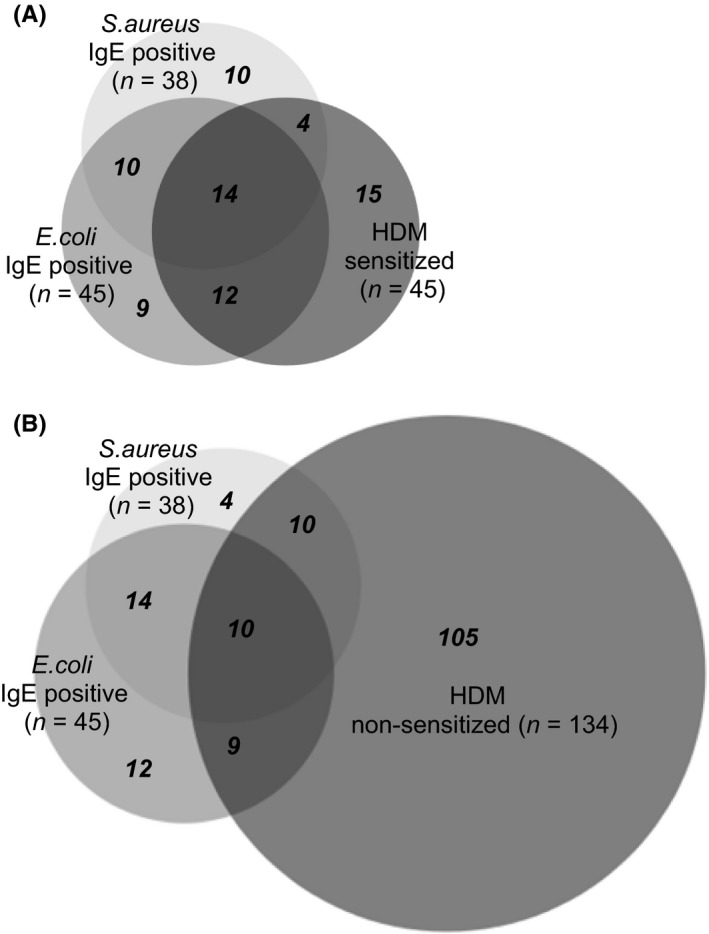
Occurrence of IgE reactivity to *Staphylococcus aureus* (*S. aureus*) and/or *Escherichia coli* (*E. coli*) antigens among Swedish AD patients with or without IgE sensitization to house dust mites (HDM) allergens. Patients with AD that had positive IgE immunoblots with either *S. aureus* extract or *E. coli* extract, or both extracts are depicted in an area‐proportional Venn diagram among AD patients (A) with or (B) without IgE sensitization to HDM allergens on MeDALL allergen chip. The numbers of patients in each of the overlapping and exclusive compartments are indicated in bold italic font

As it has been reported that the microbiome of HDM contains various bacteria, including *S. aureus* and *E. coli,* 18 we were wondering whether IgE sensitization to bacterial antigens may be associated with IgE sensitization to HDM. We therefore investigated the frequencies of sensitization to bacterial antigens in AD patients with and without HDM sensitization (Table 1). This analysis showed that 40% and 58% of HDM‐sensitized AD patients had IgE antibodies specific for *S. aureus* and *E. coli,* respectively, whereas only 15% and 14% of AD patients without HDM sensitization showed IgE reactivity to *S. aureus* and *E. coli*. IgE reactivity to *S. aureus* and/or *E. coli* was present in 67% of the HDM‐sensitized patients but only in 22% of those without HDM sensitization. The odds ratios for having IgE antibodies against *E. coli*,* S. aureus* or one of the two were 8.3, 3.8 and 7.2 in the HDM‐sensitized vs the HDM‐nonsensitized AD patients, respectively (Table 1). The detailed frequencies of bacterial IgE sensitization among the HDM‐sensitized and HDM‐nonsensitized Swedish AD patients are depicted in the form of Venn diagrams in Figure 2.

Age and gender distribution did not differ significantly between participants with bacterial IgE and those without bacterial IgE (Table S2B‐D).

### 3.4 IgE sensitization to bacterial antigens but not to HDM allergens is associated with severe AD

**Figure 3:**
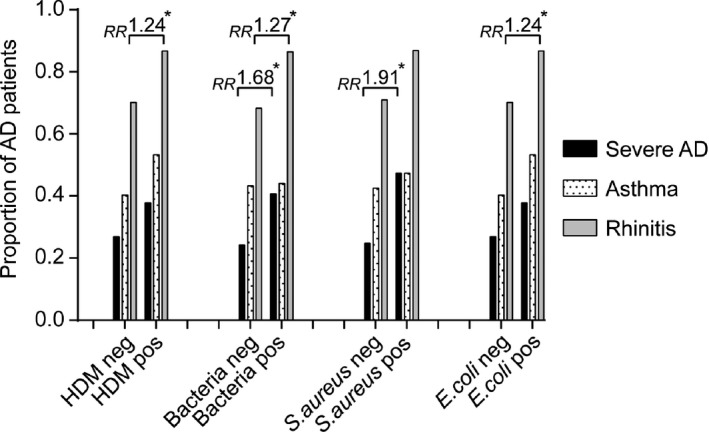
Association between IgE sensitization to *Staphylococcus aureus* (*S. aureus*) and *Escherichia coli* (*E. coli*) antigens, rhinitis and severe AD. Proportion of AD patients (*y*‐axis) with severe AD (black bars), asthma (dotted bars) or rhinitis (grey bars) in patients groups (*x*‐axis: house dust mites (HDM) IgE negative, HDM IgE positive, negative or positive for IgE against bacteria [ie, *S. aureus* and/or *E. coli*]). Relative risks (RR) are indicated above the respective bars when significant (*P < .05)

Figure 3 shows the proportion of patients with severe AD, history of rhinitis and history of asthma among patients with or without IgE reactivity to HDM allergens and bacterial antigens. Table S2 provides detailed information regarding the numbers, gender and age of the patients in the different groups and concerning significant differences regarding the presence of moderate or severe AD and history of rhinitis and asthma in the groups with HDM and bacterial IgE sensitizations. We found that IgE reactivity to HDM and to *S. aureus* and/or *E. coli* was associated with rhinitis. Having IgE against HDM or IgE specifically against *E. coli* had a relative risk of 1.24 (*P* = .0306) associated with rhinitis (Figure 3, Table S2A,D). IgE reactivity to *S. aureus* and/or *E. coli* was also associated with severe AD (ie, relative risk 1.68; *P* = .0359) (Figure 3, Table S2B). In particular, AD patients with IgE antibodies against *S. aureus* had an almost twofold increase in the relative risk (1.91; *P* = .0093) associated with severe AD compared to AD patients without IgE antibodies against *S. aureus* antigens (Figure 3, Table S2C). Interestingly, we did not find associations between asthma or severe AD and IgE sensitization to HDM allergens in this population (Figure 3, Table S2A).

### 3.5 HDM contain bacterial antigens

**Figure 4:**
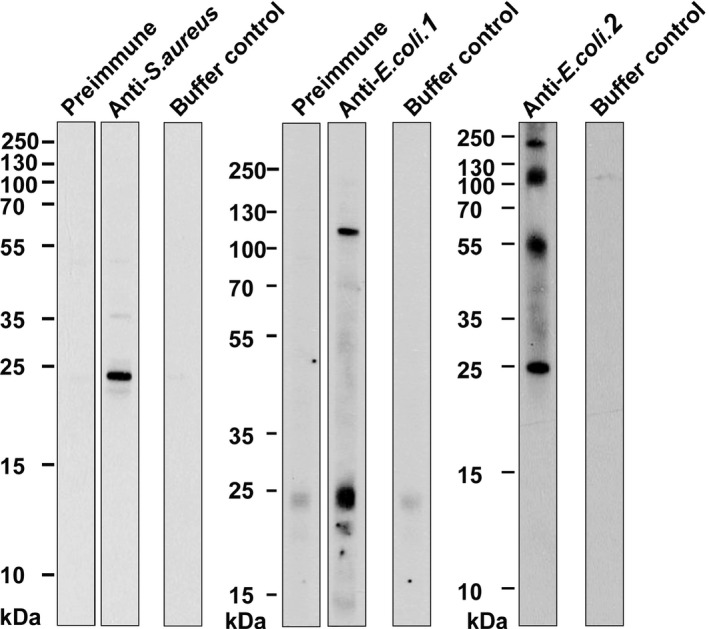
Detection of bacterial antigens or house dust mites (HDM) allergens in a HDM allergen extract. Nitrocellulose‐blotted mite body extract was tested with rabbit antibodies specific for bacterial antigens (anti‐*S. aureus*, anti‐*E. coli.1*, anti‐*E. coli.2*). Rabbit preimmune sera and/or buffer served as negative controls. Molecular weight markers are shown in kilo Daltons (kDa) on the left margins

To search for the presence of bacterial antigens in HDM, we prepared extracts from HDM bodies, separated them by SDS‐PAGE and used rabbit antisera raised against *S. aureus* extract, *E. coli* extract or *E. coli* proteins, to detect *S. aureus* and *E. coli* antigens by immunoblotting (Figure 4). The antiserum raised against *S. aureus* extract reacted specifically with a 25 kDa band in nitrocellulose‐blotted HDM extract, whereas no reactivity was observed with the preimmune rabbit serum or the buffer control (Figure 4). Likewise, antisera raised against *E. coli* extract (anti‐*E. coli.1*), and against low to high molecular weight *E. coli* proteins (anti‐*E. coli.2*), reacted with distinct bands between 20 and 250 kDa in blotted HDM extracts (Figure 4).

For control purposes, we tested rabbit sera raised against purified HDM allergens (nDer p 1, rDer p 2, rDer p 5, rDer p 7, rDer p 10, rDer p 11, rDer p 14, rDer p 21, rDer p 23 and clone 16) for reactivity to the nitrocellulose‐blotted HDM extract, *S. aureus* extract and *E. coli* extract. The antisera showed specific positive reactions with Der p 2, Der p 5, Der p 7, Der p 10, Der p 11 and Der p 21 in the blotted HDM extract, and preimmune sera did not show positive reactions (Figure S2A). No relevant reactivity over background (ie, preimmune sera, buffer control) was observed when the rabbit antisera were tested with blotted *S. aureus* and *E. coli* extracts (Figure S2B,C).

### 3.6 HDM contain IgE‐reactive bacterial antigens

**Figure 5:**
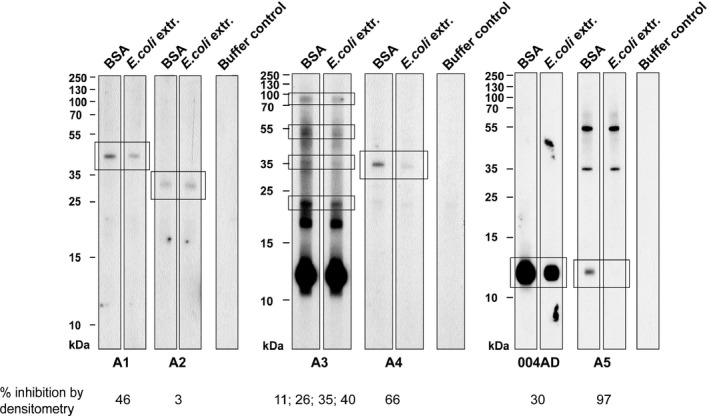
Pre‐incubation of plasma/serum samples from patients (A1‐A5, 004AD) with IgE reactivity to bacterial antigens with an *Escherichia coli* (*E. coli*) extract inhibits IgE binding to distinct antigens in blotted house dust mites (HDM) extracts. IgE reactivity of plasma/serum samples which had been preincubated with *E. coli* extract or BSA (negative control) to nitrocellulose‐blotted HDM extracts. Buffer controls represent buffer without addition of plasma/serum. Bands that were inhibited by adsorption with *E. coli* are boxed, and the % reduction determined by densitometry in band density is given below. For subject A3, % reductions are shown from top to bottom. Molecular weight markers are shown in kilo Daltons (kDa) on the left margins

To detect IgE‐reactive bacterial antigens in HDM, we then performed IgE immunoblot inhibition experiments^39^ using serum/plasma samples where sufficient volumes were available. We tested whether preadsorption of plasma/sera from patients with AD with *E. coli* extract can inhibit IgE binding to blotted HDM extract (Figure 5). Table S1 provides a description of the clinical features and IgE reactivity profiles of the patients used in the IgE inhibition experiments. Two of these patients (A3 and 004AD, Table S1) showed high IgE levels to Der p 2 at approximately 13 kDa (Figure 5), which is well represented in HDM extracts.^20^, ^36^ Patient A5 showed only very low IgE levels to Der p 2. Patients A1, A2, A3 and A5 showed IgE reactivity to Der p 23 which is poorly represented in extracts obtained from HDM bodies,^36^ and patient A4 (Table S1) did not show IgE reactivity to any of the purified HDM allergens tested. For five of the six tested patients (ie, A1, A3, A4, 004AD, A5), we found that preadsorption of the sera with *E. coli* inhibited IgE binding to distinct bands in blotted HDM extracts (Figure 5). The inhibition of IgE binding was highly specific as exemplified for patient A5 where preadsorption with *E. coli* inhibited IgE binding to a 12 to 13 kDa band but did not affect IgE reactivity to a 35 kDa and 55 kDa band.

In summary, the IgE inhibition experiments identified bands with molecular weights of approx. 40 kDa (A1), 23 kDa, 35 kDa, 55 kDa and 90 kDa (A3), 35 kDa (A4) and 12‐13 kDa (004AD, A5) which were inhibited with *E. coli* in blotted HDM extracts (Figure 5). The extent of inhibition according to densitometry analysis of the bands ranged from 3% to 97% (Figure 5).

### 3.7 Quantification of IgE towards bacterial antigens in HDM extracts

**Figure 6:**
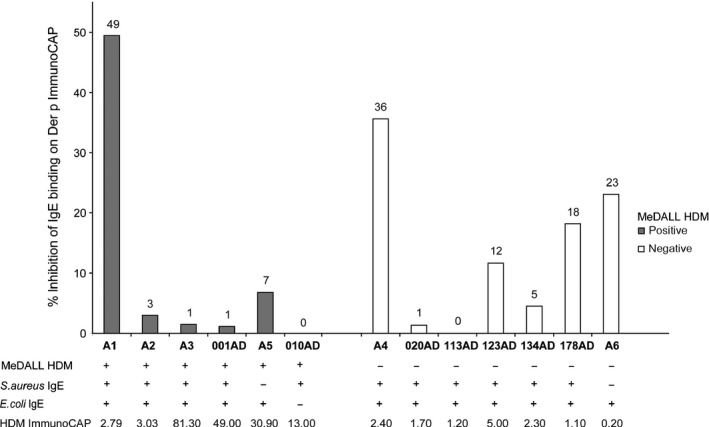
Preincubation of plasma/serum samples from patients with IgE antibodies to bacterial antigens, with *Escherichia coli* (*E. coli*) extract inhibits IgE binding to house dust mites (HDM) extract when measured by Der p ImmunoCAP™ assay. The percentages of inhibition of IgE binding (*y*‐axes, and indicated above the bars) obtained by preadsorption of samples with an *E. coli* extract are shown for patients with (left, grey bars) and without (right, white bars) IgE sensitization to HDM allergens. IgE reactivities to HDM allergens determined by allergen chip, *S. aureus* and *E. coli* extracts determined by immunoblotting, as well as IgE levels against HDM extract determined by Der p ImmunoCAP™ are shown for each patient at the bottom of the figure

The IgE immunoblot inhibition experiments demonstrated that extracts from HDM contain IgE‐reactive bacterial antigens. We were therefore interested to quantify the IgE levels towards bacterial antigens by ImmunoCAP™ inhibition experiments. Figure 6 shows the percentages of inhibition of IgE binding, which could be obtained when patients’ plasma/sera (Figure 6, Table S1) were preadsorbed with *E. coli* extract and the remaining IgE towards HDM was determined by Der p ImmunoCAP™ measurements. We found that preadsorption of plasma/serum samples with *E. coli* extract inhibited IgE binding to HDM to varying extents and reached almost 50% (percentages inhibition: 0%‐49%). For control purposes, we included a plasma sample from a patient (010AD) who was positive for IgE binding only to *S. aureus* but not to *E. coli* (Table S1). No reduction in IgE binding was observed in this sample after preincubation with *E. coli* extract (Figure 6).

## 4 DISCUSSION

HDM not only represent one of the most potent and frequent allergen sources worldwide, but they also contain factors that alter barrier function, induce pro‐inflammatory cytokines and affect IgE responses as well as the innate immune system.^25^ Recently, it has been reported that the HDM microbiome contains a variety of Gram‐positive and Gram‐negative bacteria including *S. aureus* and *E. coli*.^18^ These bacteria seem to be important as part of the HDM microbiome because mites do not thrive when cultured with antibiotics,^45^ suggesting that bacteria exist as real endosymbionts of HDM and are not mere contaminants. In this study, we have investigated the hypothesis that HDM may have another important hitherto unknown function in allergy, namely to act as carriers for bacteria and thus participate in IgE sensitization to bacterial antigens. In fact, IgE sensitization to bacteria is common in respiratory and skin manifestations of allergy,^2^, ^3^, ^4^, ^5^, ^6^, ^7^, ^13^, ^15^ but the mechanism of sensitization, particularly to Gram‐negative bacteria such as *E. coli,* has been unknown.

We provide evidence that HDM are indeed carriers for IgE sensitization to bacterial antigens via two major approaches. First, we have investigated a well‐defined group of patients with AD from Sweden, a country where HDM allergy is less frequent compared to other countries due to the dry and cold temperate climate.^26^ We therefore could compare the frequencies of IgE sensitizations to bacterial antigens in HDM‐sensitized and HDM‐nonsensitized AD patients. The investigation of the frequency of sensitization to HDM allergens and bacterial antigens from *S. aureus* and *E. coli* showed that IgE sensitization to bacterial antigens was significantly more frequent in HDM‐sensitized patients compared to HDM‐nonsensitized patients.

Interestingly, bacterial IgE sensitization was associated with rhinitis as comorbidity, indicating the possible clinical relevance of IgE sensitization to bacterial antigens. Another interesting finding was that we observed IgE sensitization to both *S. aureus* and *E. coli* antigens in more than 50% of patients with bacterial sensitizations, which could support the possibility of a common route and mechanism of sensitization through a carrier such as HDM. Second and most important, we could demonstrate the presence of bacterial antigens in purified HDM bodies using specific antibody probes and verify that HDM contain IgE‐reactive bacterial antigens by qualitative IgE immunoblot and quantitiative ImmunoCAP inhibition experiments. As none of the antibody probes specific for known HDM allergens reacted with the bacterial antigens and BLAST analysis of the mite allergens showed no sequence homology with bacteria, it is unlikely that the observations we have made are due to cross‐reactivity between HDM and bacterial antigens.

These experiments thus demonstrate that HDM indeed contain IgE‐reactive bacterial antigens. An interesting finding made in the context of the IgE inhibition experiments was that certain patients who exhibited rather low levels of IgE towards HDM extracts showed no IgE reactivity to the panel of purified HDM allergens (Fig. 6, Table [Link], [Link], [Link]) which in previous studies has been shown to identify bona fide most of the HDM‐sensitized patients.^40^, ^41^, ^42^ In fact, we found that a considerable percentage of IgE which seemed to be specific for HDM allergen extract could be preadsorbed with bacterial antigens indicating that these patients were sensitized to bacteria rather than to HDM. Therefore, the possibility that IgE sensitization to bacterial antigens may cause a false‐positive test result with HDM extracts containing bacteria when diagnostic tests are performed with HDM extract needs to be considered.

In a previous study from our group,^15^ we addressed the question whether IgE reactivity to bacterial antigens is due to high total IgE levels, and we found that high IgE levels were not a prerequisite for reactivity to bacterial antigens. In our current study, we observed that even though elevated total plasma IgE levels were associated with IgE reactivity to either *S. aureus* or *E. coli,* not all AD patients with high IgE showed reactivity to the bacterial extracts (*S. aureus* IgE‐negative sample with IgE 2800 kU/L; *E. coli* IgE‐negative sample with IgE 10 600 kU/L), and some patients with relatively low total IgE levels had IgE to bacteria (*S. aureus* IgE‐positive sample with IgE 38 kU/L; *E. coli* IgE‐positive sample with IgE 150 kU/L). Elevated total plasma IgE is therefore not a prerequisite for IgE binding to bacterial antigens.

IgE sensitization to bacterial antigens, particularly from *S. aureus,* has been shown to be clinically relevant in respiratory allergy and AD.^3^, ^5^, ^14^ Sensitization to Gram‐negative bacteria (eg, *H. influenzae*) may also have protective activity,^2^, ^4^, ^46^ but the clinical relevance of IgE sensitization to *E. coli* has not been investigated so far. It is, however, possible that IgE against *E. coli* may play a role in gastrointestinal manifestations that mimic food allergy, and such gastrointestinal disturbances are quite common in patients with AD.^47^, ^48^, ^49^

In summary, the results of our study demonstrate that HDM can act as a carrier for antigens from bacteria which colonize the skin, the respiratory tract and the gut and thus may cause sensitization to bacterial antigens. This could explain the frequent occurrence of IgE reactivity to bacterial antigens in respiratory and skin manifestations of allergy.

## CONFLICTS OF INTEREST

Rudolf Valenta has received research grants from Biomay AG, Vienna, Austria, and Viravaxx, Vienna, Austria. He serves as a consultant for Biomay AG, Viravaxx, Fresenius Medical Care, Bad Homburg, Germany, and for Boehringer Ingelheim, Biberach, Germany. The other authors have no conflict of interest to declare.

## AUTHOR CONTRIBUTIONS

SD, IM, YR‐M performed experiments, analysed data, wrote manuscript and read the manuscript; SV, MN, and AMH, analysed data and read the manuscript. GW and LL contributed with clinical data and samples from the Swedish cohort and read the manuscript; CJ and AS analysed data, wrote parts of the manuscript and read the manuscript; RV designed and supervised experiments, analysed data, wrote manuscript and read the manuscript.
