# Supplementary figures and images for: House dust mites as potential carriers for IgE sensitization to bacterial antigens
Source: Allergy. 2017 Sep 7;73(1):115–24. doi: 10.1111/all.13260 (PMC5763376; doi:10.1111/all.13260)

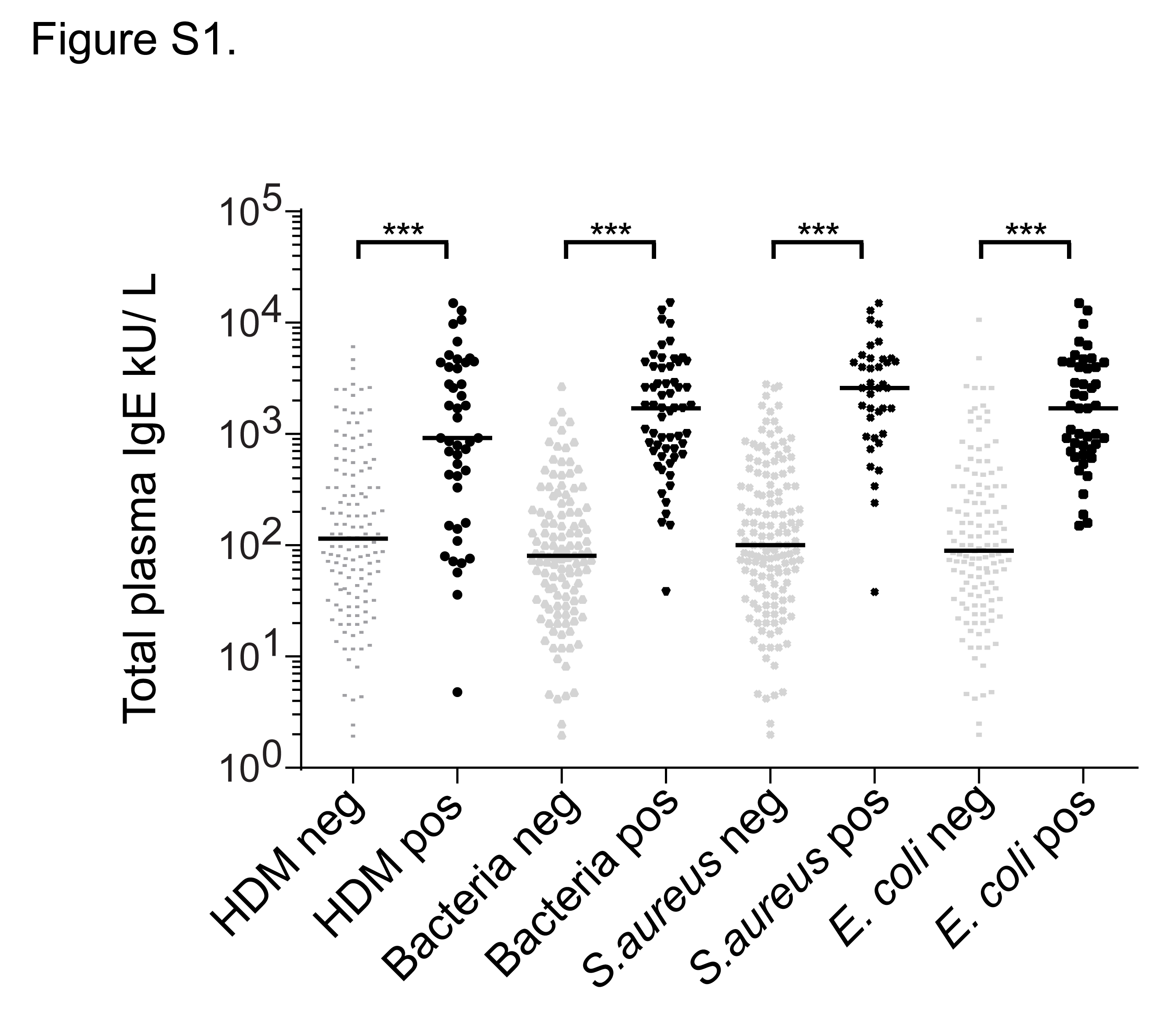

Supplement: Supplementary file 1 [file ALL-73-115-s001.tif]

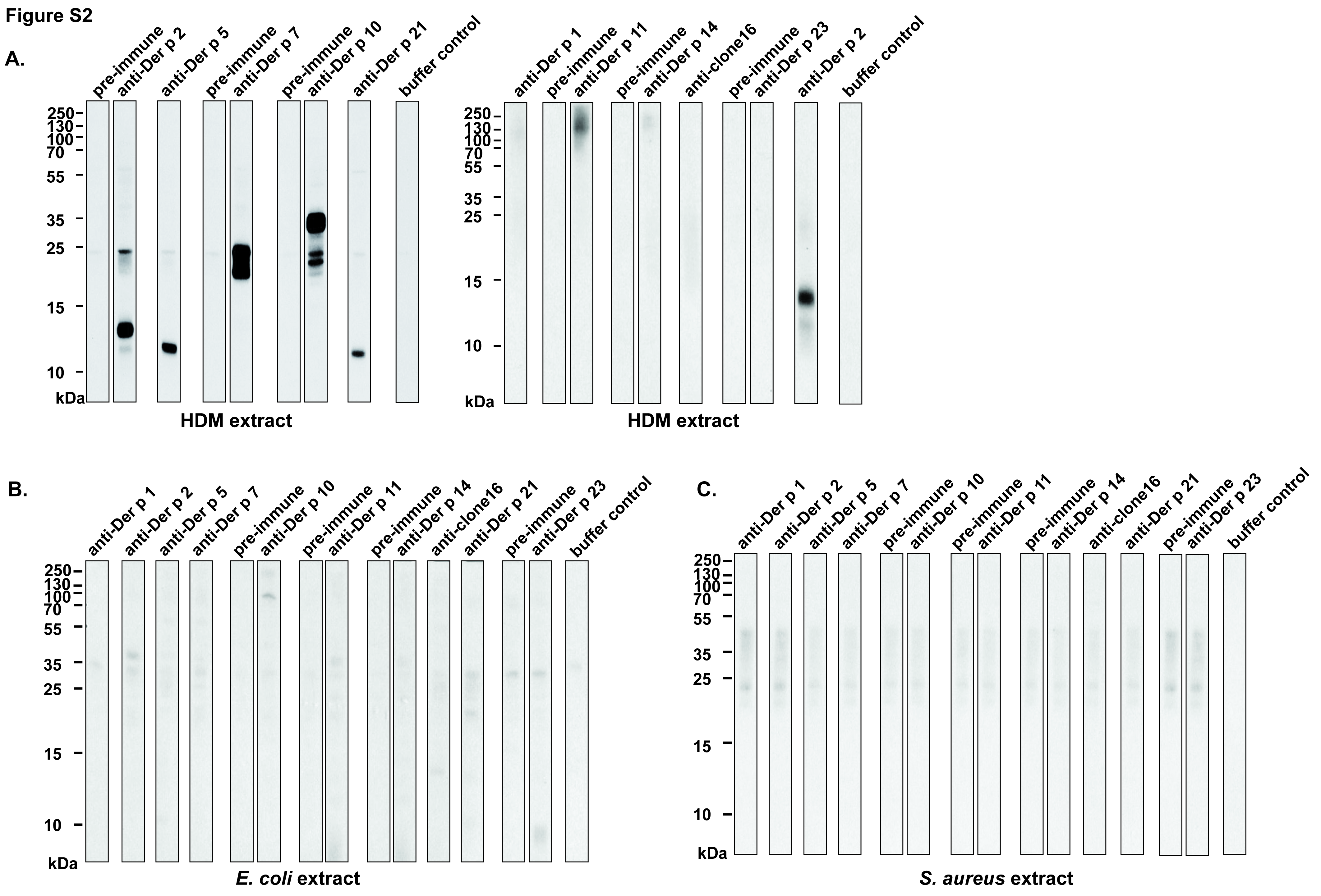

Supplement: Supplementary file 2 [file ALL-73-115-s002.tif]

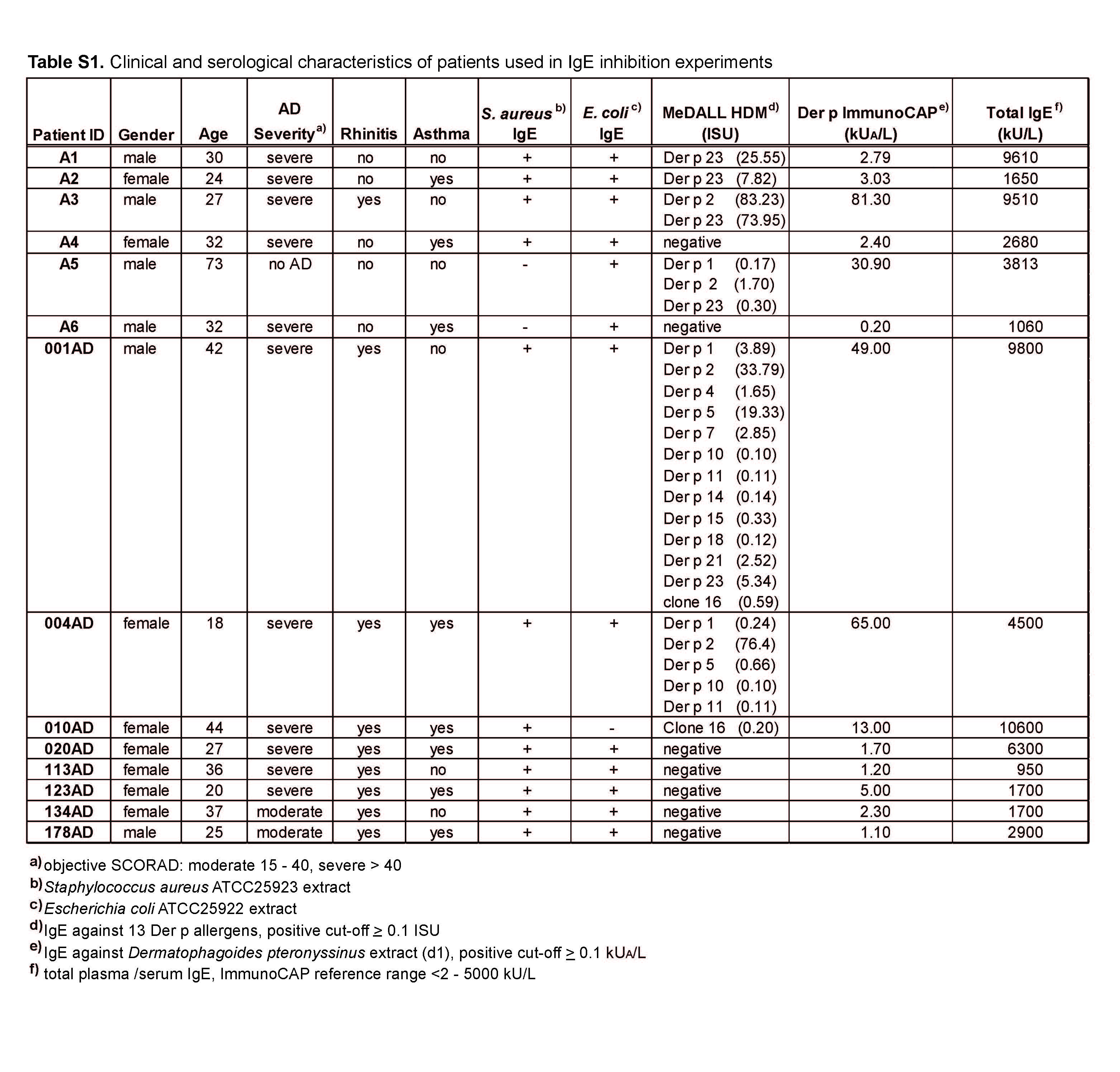

Supplement: Supplementary file 3 [file ALL-73-115-s003.jpg]

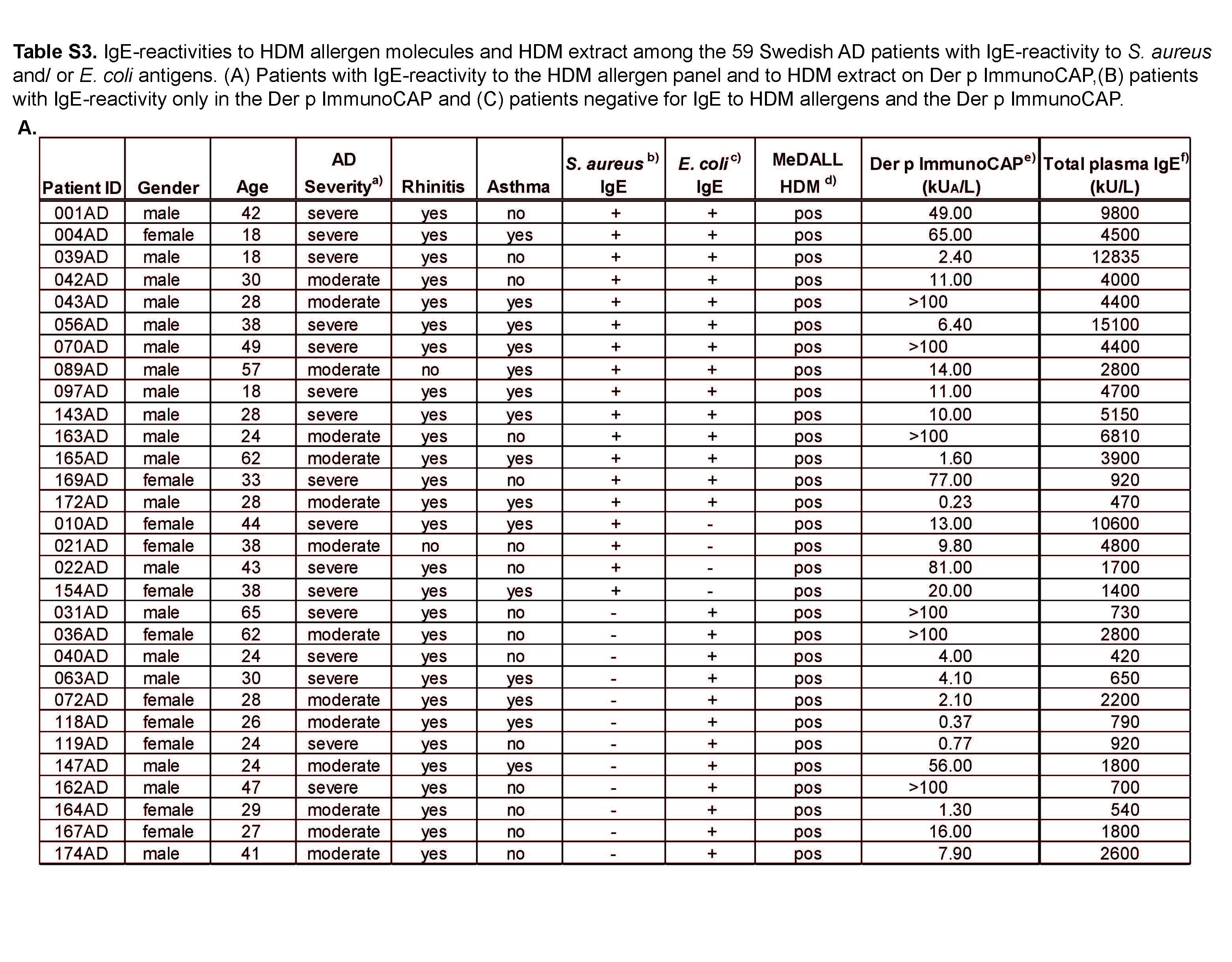

Supplement: Supplementary file 5 [file ALL-73-115-s005.jpg]

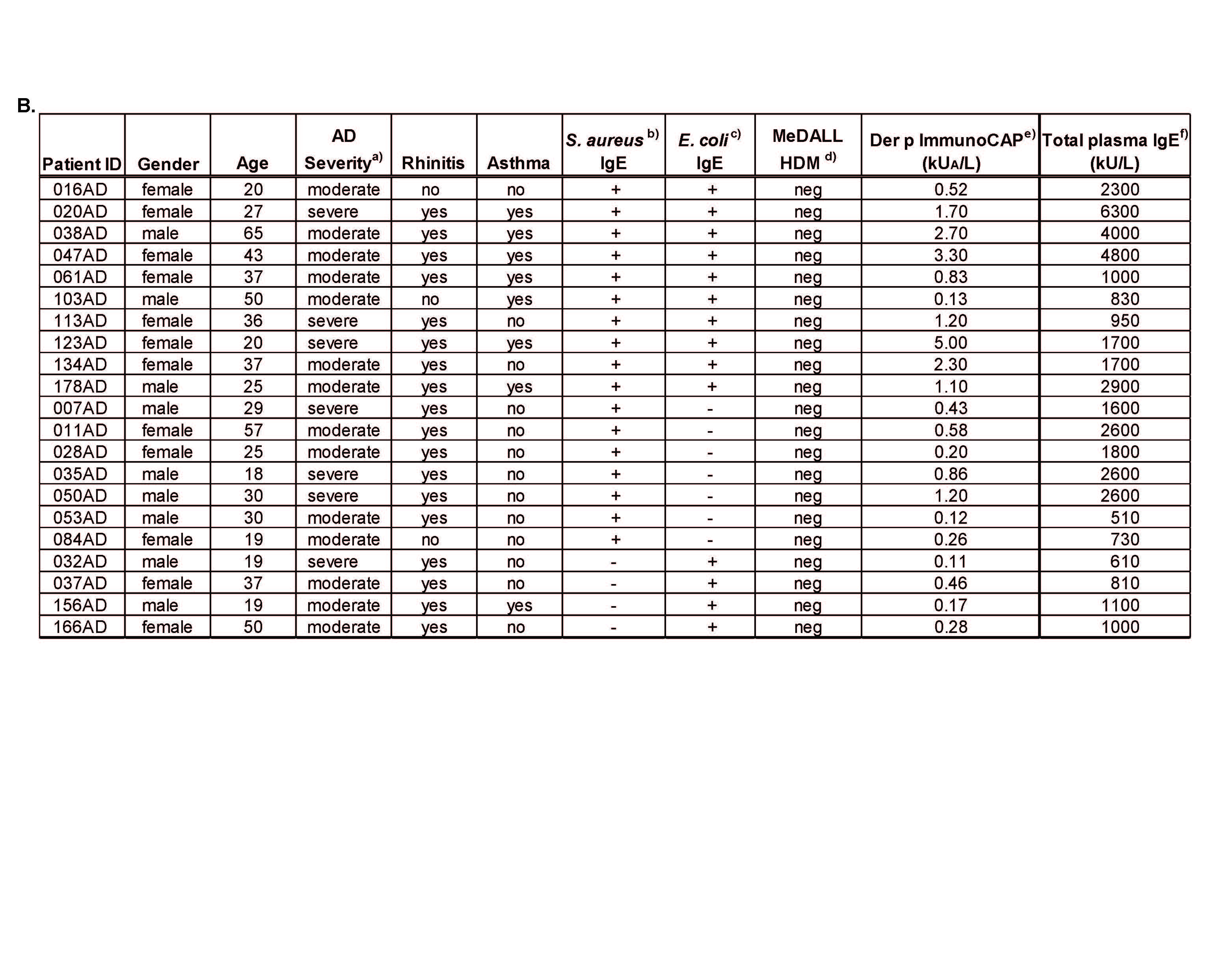

Supplement: Supplementary file 6 [file ALL-73-115-s006.jpg]

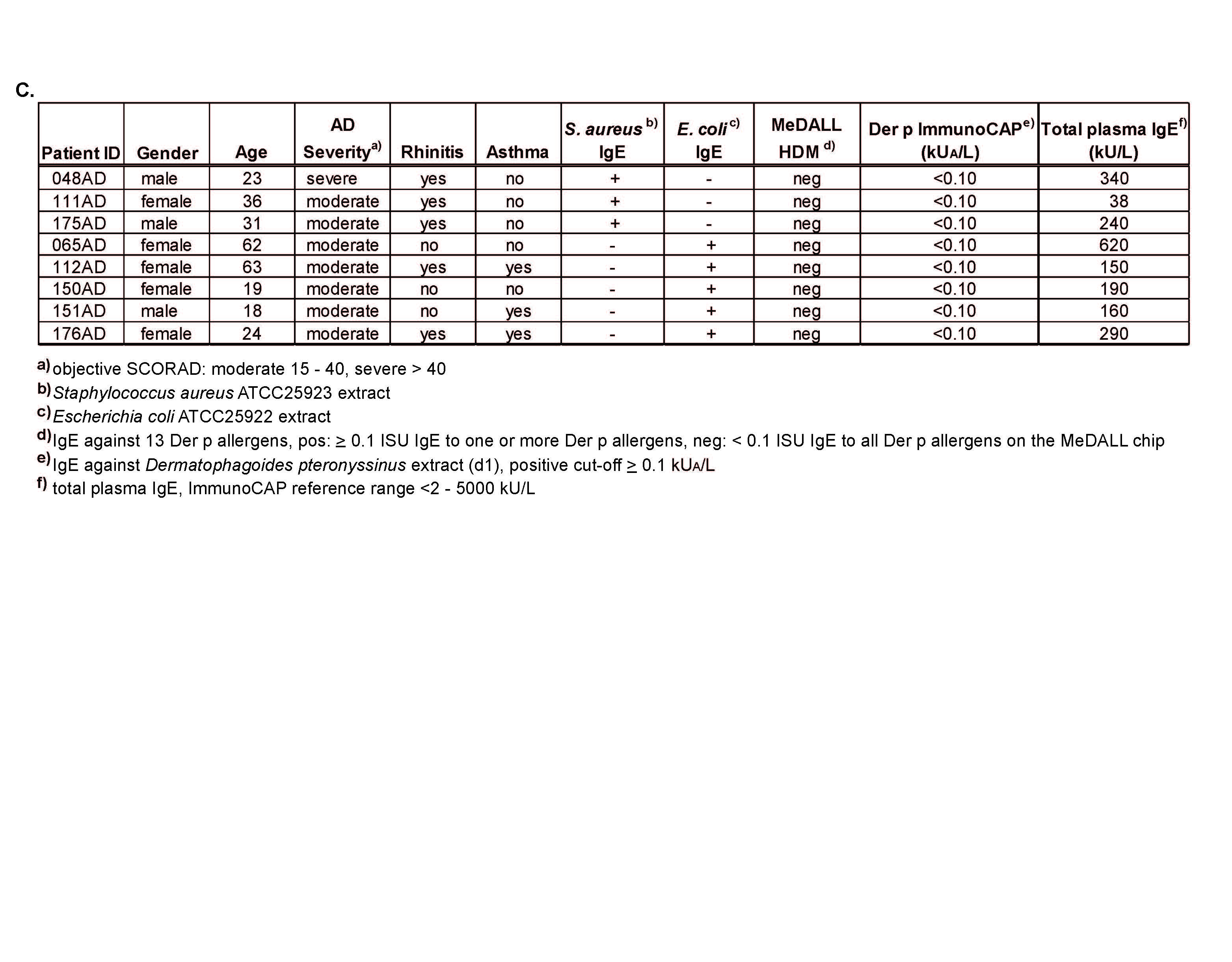

Supplement: Supplementary file 7 [file ALL-73-115-s007.jpg]
