# Supplementary material for: House dust mites as potential carriers for IgE sensitization to bacterial antigens
Source: Allergy. 2017 Sep 7;73(1):115–24. doi: 10.1111/all.13260 (PMC5763376; doi:10.1111/all.13260)
Supplement: Supplementary file 4 [file ALL-73-115-s004.doc]

**Table S2.** Demographic and clinical data of the AD patients in the Swedish cohort, after stratification by IgE reactivity to **(A)** HDM allergens, **(B)** *S. aureus* and/ or *E. coli*, **(C)** *S. aureus* or **(D)** *E. coli*.

**A.**

IgE specific to HDM allergens ^a)^

positive (n=45) negative (n=134)

Characteristic n (%) n (%) statistic (95% CI) p-value

Males (n=78) 23 (51) 55 (41)

Females (n=101) 22 (49) 79 (59) *_P1-P2_* 0.1007 (-0.06682 to 0.2681) 0.2974

Median age in years (range) 30 (18 – 65) 27 (18 – 65) *_M1-M2_* -3.000 (-6.000 to 1.000) 0.1374

History of rhinitis (n=133) 39 (87) 94 (70) *_RR_* 1.235 (1.0540 to 1.449) 0.0306*

History of asthma (n=78) 24 (53) 54 (40) *_RR_* 1.323 (0.9398 to 1. 864) 0.1644

Moderate AD ^b)^ (n=126) 28 (62) 98 (73)

Severe AD ^c)^ (n=53) 17 (38) 36 (27) *_RR_* 1.406 (0.8809 to 2.245) 0.1883 Median total IgE kU/L ^d)^ (range) 920 (4.8 – 15100) 115 (<2 – 6300) *_M1-M2_* 805 (410 to 1510) <0.0001^***^

**B.**

IgE specific to *S. aureus* and/ or *E. coli* ^e)^

positive (n=59) negative (n=120)

Characteristic n (%) n (%) statistic (95% CI) p-value

Males (n=78) 31 (53) 47 (39)

Females (n=101) 28 (47) 73 (61) *_P1-P2_* 0.1338 (-0.0208 to 0.2883) 0.1092

Median age in years (range) 30 (18 – 65) 27 (18 -63) *_M1-M2_* -3.000 (-6.000 to 2.000) 0.2998

History of rhinitis (n=133) 51 (86) 82 (68) *_RR_* 1.265 (1.080 to 1.482) 0.0105^*^

History of asthma (n=78) 26 (44) 52 (43) *_RR_* 1.017 (0.7145 to 1.447) 1.0000

Moderate AD ^b)^ (n=126) 35 (59) 91 (76)

Severe AD ^c)^ (n=53) 24 (41) 29 (24) *_RR_* 1.683 (1.082 to 2.619) 0.0359^*^

Median total IgE kU/L ^d)^ (range) 1700 (38 – 15100) 80.5 (<2 – 2700) *_M1-M2_* 1620 (904 to 1940) <0.0001^***^

**C.**

IgE specific to *S. aureus*

positive (n=38) negative (n=141)

Characteristic n (%) n (%) statistic (95% CI) p-value

Males (n=78) 22 (58) 56 (40)

Females (n=101) 16 (42) 85 (60) *_P1-P2_* 0.1818 (0.0041 to 0.3595) 0.0645

Median age in years (range) 31 (18 - 65) 27 (18 - 65) *_M1-M2_* -3.500 (-6.000 to 2.000) 0.3949

History of rhinitis (n=133) 33 (87) 100 (71) *_RR_* 1.224 (1.041 to 1.441) 0.0590

History of asthma (n=78) 18 (47) 60 (43) *_RR_* 1.113 (0.7565 to 1.638) 0.7128

Moderate AD ^b)^ (n=126) 20 (53) 106 (75)

Severe AD ^c)^ (n=53) 18 (47) 35 (25) *_RR_* 1.908 (1.227 to 2.967) 0.0093^*^

Median total IgE kU/L ^d)^ (range) 2600 (38 – 15100) 100 (<2 – 2800) *_M1-M2_* 2500 (1640 to 3390) <0.0001^***^

**D.**

IgE specific to *E. coli*

positive (n=45) negative (n=134)

Characteristic n (%) n (%) statistic (95% CI) p-value

Males (n=78) 24 (53) 54 (40)

Females (n=101) 21 (47) 80 (60) *_P1-P2_* 0.1303 (-0.0371 to 0.2978) 0.1644

Median age in years (range) 29 (18 - 65) 28 (18 - 63) *_M1-M2_* -1.500 (-6.000 to 2.000) 0.3914

History of rhinitis (n=133) 39 (87) 94 (70) *_RR_* 1.235 (1.054 to 1.449) 0.0306^*^

History of asthma (n=78) 24 (53) 54 (40) *_RR_* 1.323 (0.9398 to 1.864) 0.1644

Moderate AD ^b)^ (n=126) 28 (62) 98 (73)

Severe AD ^c)^ (n=53) 17 (38) 36 (27) *_RR_* 1.406 (0.8809 to 2.245) 0.1883

Median total IgE kU/L ^d)^ (range) 1700 (150 – 15100) 89.5 (<2 – 10600) *_M1-M2_* 1611 (814 to 2124) <0.0001^***^

1. IgE level ≥ 0.1 ISU, to at least one of 13 Der p allergens on the MeDALL chip
2. objective SCORAD 15 - 40
3. objective SCORAD > 40
4. total plasma IgE measured on total IgE ImmunoCAP^TM^, reference range <2 – 5000 kU/L
5. *Staphylococcus aureus* ATCC25923 extract, *Escherichia coli* ATCC25922 extract

CI - confidence interval; *_P1-P2_* – difference between proportions; *_M1-M2_* – difference between medians; *_RR_* – relative risk (risk ratio)

^*^- p< 0.05; ^***^- p< 0.0001
